# Risk Factors for Contra-Lateral Secondary Anterior Cruciate Ligament Injury: A Systematic Review with Meta-Analysis

**DOI:** 10.1007/s40279-020-01424-3

**Published:** 2021-01-30

**Authors:** Anna Cronström, Eva Tengman, Charlotte K. Häger

**Affiliations:** 1 Department of Community Medicine and Rehabilitation, Umeå University, Umeå, Sweden; 2 Department of Health Sciences, Lund University, Lund, Sweden

## Abstract

**Background:**

The risk of sustaining a contra-lateral anterior cruciate ligament (C-ACL) injury after primary unilateral ACL injury is high. C-ACL injury often contributes to a further decline in function and quality of life, including failure to return to sport. There is, however, very limited knowledge about which risk factors that contribute to C-ACL injury.

**Objective:**

To systematically review instrinsic risk factors for sustaining a C-ACL injury.

**Methods:**

A systematic review with meta-analysis was performed according to the Preferred Reporting Items for Systematic Reviews and Meta-Analyses guidelines. Four databases (MEDLINE, CINAHL, EMBASE, Sport Discus) were searched from inception to January 2020. Inclusion criteria were prospective or retrospective studies investigating any intrinsic risk factor for future C-ACL injury. Meta-analysis was performed and expressed as odds ratios (OR) if two or more articles assessed the same risk factor.

**Results:**

44 moderate-to-high quality studies were eventually included in this review, whereof 35 studies were eligible for meta-analysis, including up to 59 000 individuals. We identified seven factors independently increasing the odds of sustaining a C-ACL injury (in order of highest to lowest OR): (1) returning to a high activity level (OR 3.26, 95% CI 2.10–5.06); (2) Body Mass Index < 25 (OR 2.73, 95% CI 1.73–4.36); (3) age ≤ 18 years (OR 2.42, 95% CI 1.51–3.88); (4) family history of ACL injury (OR 2.07, 95% CI 1.54–2.80); (5) primary ACL reconstruction performed ≤ 3 months post injury (OR 1.65, 95% CI: 1.32–2.06); (6) female sex (OR 1.35, 95% CI 1.14–1.61); and (7) concomitant meniscal injury (OR 1.21, 95% CI 1.03–1.42). The following two factors were associated with decreased odds of a subsequent C-ACL injury: 1) decreased intercondylar notch width/width of the distal femur ratio (OR 0.43, 95% CI 0.25–0.69) and 2) concomitant cartilage injury (OR 0.83, 95% CI 0.69–1.00). There were no associations between the odds of sustaining a C-ACL injury and smoking status, pre-injury activity level, playing soccer compared to other sports or timing of return to sport. No studies of neuromuscular function in relation to risk of C-ACL injury were eligible for meta-analysis according to our criteria.

**Conclusion:**

This review provides evidence that demographic factors such as female sex, young age (≤ 18 years) and family history of ACL injury, as well as early reconstruction and returning to a high activity level increase the risk of C-ACL injury. Given the lack of studies related to neuromuscular factors that may be modifiable by training, future studies are warranted that investigate the possible role of factors such as dynamic knee stability and alignment, muscle activation and/or strength and proprioception as well as sport-specific training prior to return-to-sport for C-ACL injuries.

**PROSPERO**: CRD42020140129.

**Supplementary Information:**

The online version contains supplementary material available at 10.1007/s40279-020-01424-3.

## Key Points

**Table:** 

Returning to a high activity level was the risk factor with the highest odds for sustaining a contra-lateral anterior cruciate ligament (C-ACL) injury following primary unilateral ACL injury.	
In addition, females, individuals younger than 18 years, those with a family history of ACL injury and those receiving primary reconstruction within 3 months of injury had an increased risk of C-ACL.	
Very few studies were identified investigating the potential influence of modifiable factors, including muscle strength, movement patterns and knee stability on the risk of C-ACL injury.	

## Background

Anterior cruciate ligament (ACL) injury is a common sports-related injury [1, 2] which often results in functional limitations and a lower activity level that may persist over time [3–6].

Indeed, many ACL-injured individuals never return to their pre-injury activity level [7]. Additionally, the risk of sustaining a subsequent contra-lateral ACL (C-ACL) injury is high and has even been reported to be significantly higher than suffering a new injury to the ipsi-lateral knee [8, 9]. A systematic review with meta-analysis from 2016 calculated the overall C-ACL injury rate to be around 8%, and even higher (12%) in younger individuals involved in sports at an elite level [9]. Sustaining a C-ACL injury is associated with even further decline in function and quality of life, lower level of activity and increased risk of failure to return to sport/activity compared to unilateral injury [10]. Nevertheless, little attention has been given to the matter in research and the different factors that may pre-dispose some individuals for subsequent ACL injuries remain unclear [11].

Previous research has established several risk factors for primary ACL injury, such as female sex [1], increased joint laxity [12], BMI [13, 14], family history [15], reduced lower extremity strength [16] and altered trunk and knee biomechanics [16]. However, given that individuals with ACL injury exhibit altered sensorimotor function, such as reduced lower extremity strength [17], altered biomechanics [18] and impaired neuromuscular control [17] compared to non-injured individuals, the risk factors for C-ACL injury may be entirely different from those associated with primary injury. Currently, there is some evidence identifying younger age and higher activity level as risk factors for subsequent ACL injury to either knee [9], whereas conflicting results have been reported for sex, family history and geometrics as risk factors for C-ACL injury [11]. To our knowledge, all potential instrinsic (patient-related) risk factors for C-ACL injury have never been systematically synthezised. To identify the factors that pre-dispose individuals to C-ACL injury after primary ACL injury is important for screening of injury risk as well as for optimizing training and rehabilitation after ACL injury to minimize the risk of re-injury and associated consequences for these individuals. Thus, the aim of this study was to systematically review instinsic risk factors related to demographics, biomechanics, geometrics and function, that could each be independently associated with sustaining a C-ACL injury.

## Methods

This systematic review with meta-analysis was conducted according to the Preferred Reporting Items for Systematic Reviews and Meta-Analyses (PRISMA) guidelines [19, 20]. The study protocol was pre-registered (PROSPERO: CRD42020140129; n.b. registration and search terms reflect a larger review initially also including graft rupture).

### Literature Search and Study Selection

A systematic search in MEDLINE (PubMed), CINAHL, EMBASE and Sport Discus was performed from inception to June 2019 and updated in January 2020 using the following terms:

#### Search Strategy

(anterior cruciate ligament [MeSH Terms] OR anterior cruciate ligament reconstruction [MeSH Terms] OR anterior cruciate ligament injury [MeSH Terms] OR “lower extremity” [Title/Abstract] OR “ACL injur*” [Title/Abstract] OR “anterior cruciate ligament injur*” [Title/Abstract] OR construct* [Title/Abstract]) AND (“risk factor*” [Title/Abstract] OR “injury risk” [Title/Abstract] OR “associated with” [Title/Abstract] OR predict* [Title/Abstract] OR relat* AND (“graft injur*” [Title/Abstract] OR “second* injur*” [Title/Abstract] OR reinjur*[Title/Abstract] OR re-injur* [Title/Abstract] OR rupture* [Title/Abstract] OR “graft failure*” [Title/Abstract]) OR “contralateral injur*” [Title/Abstract] OR “contra-lateral injur*” [Title/Abstract]).

In CINAHL, EMBASE and Sport Discus the search was performed without MeSH-terms. In addition, reference lists of all relevant articles were hand-searched for additional studies. The search was not restricted to any publication date.

#### Eligibility Criteria

Criteria for studies to be included were: (1) prospective or retrospective studies with a follow-up of any duration; (2) inclusion of males and/or females of any age with ACL injury treated with or without reconstruction; (3) assessment of any intrinsic factor (e.g. demographics, geometrics, function) at baseline; and (4) recording of at least 3 C-ACL injuries during the study period. Exclusion critera were: (1) animal studies and in vitro studies; (2) case studies, conference abstracts, review papers and editorials; (3) external risk factors (e.g. playing surface) or possible risk factors related to type of graft and/or surgery technique; or (4) published in a language other than English or Scandinavian language.

### Data Extraction and Synthesis

Two researchers (AC and ET) independently screened the titles, abstracts and full papers according to the inclusion/exclusion criteria. Any disagreements were resolved by a consensus discussion between AC and EA, and if required with a third researcher (CH). The following data were extracted from the studies: authors, publication date, number of participants, sex, age, activity level, proportion of participants sustaining a C-ACL injury, follow-up period (years), assessed risk factor (i.e. demographic, biomechanical, functional) and effect size (odds ratio). If data were not sufficiently reported in the studies, study authors were contacted and additional information was requested. A meta-analysis was performed if there were two or more studies that included the same factor, e.g. age or sex as a possible risk factor for C-ACL injury as well as metrics possible to calculate to odds ratio.

Comprehensive Meta-Analysis software, version 2.2.064 (Englewood, USA) was used for meta-analyses. The effect size was calculated as the odds ratio (95% CI) for each risk factor for sustaining a C-ACL injury. If an odds ratio was not provided in the studies, the odds ratio was calculated from the number of events and sample size. A random effect model was used due to expected heterogeneity between studies, such as sex, age, follow-up duration and activity level. Between-studies effect size heterogeneity was calculated with the *Q*-test and expressed as *I*^2^-statistics. A *p* value equal to or less than 0.05 was considered statistically significant. For studies reporting associated meniscal injuries and different types of meniscal surgeries as risk factors for C-ACL, the results for any meniscal injury (medial or lateral injury) and “any meniscal surgery” (different types of surgery pooled) were included in the meta-analysis. If medial and lateral injury was only reported separately, the result for the side (medial/lateral) included in most studies were included in the analysis and if equal, the medial side was chosen due to being the side most frequently injured [21]. Subgroup analysis for pediatrics, defiend as age < 19 years, were performed if more than one study investigated the same factor for that particular subgroup. Furthermore, sensitivity analyses for different aspects of the studies, e.g., participants’ age and follow-up duration, were performed if there were ≥ 3 studies that assessed the same risk factor.

### Quality Assessment and Publication Bias

An adapted version [22, 23] of the checklist by Downs and Black [24] (online resource 1) was used for assessment of methodological quality of the included studies by two independent reviewers (AC and ET). Any disagreements were resolved by a consensus discussion between these two reviewers and if not resolved, with a third researcher (CH). Publication bias was explored using funnel plots with trim and fill [25] if the analysis included at least ten studies [26].

## Results

**Fig. 1:**
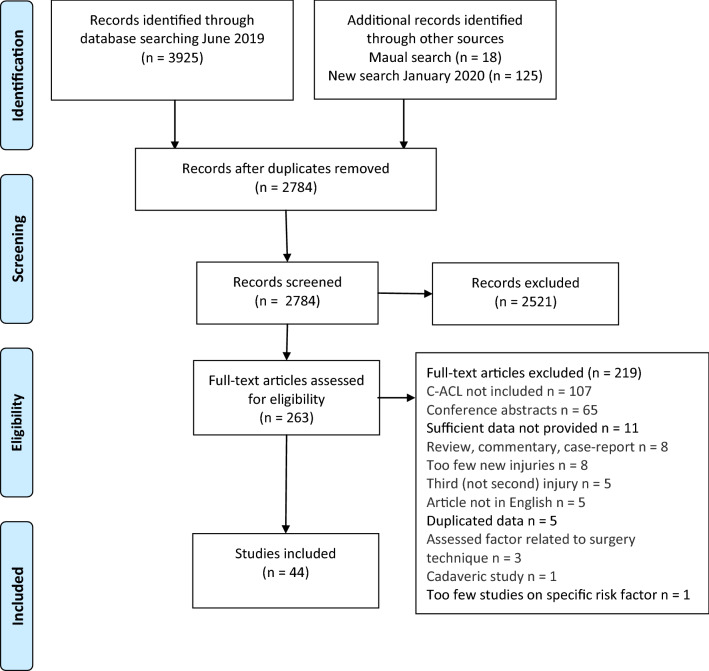
Flow chart of the inclusion process

A total of 2784 abstracts were screened according to the inclusion/exclusion criteria and 263 full-text articles were subsequently screened. 202 of those were excluded due to failure to meet the inclusion criteria. 15 articles [27–41] pooled graft rupture and C-ACL injury as second injuries or stratified their result according to different graft types instead of C-ACL injury/no C-ACL injury. The authors of these articles were contacted and additional data specifically related to C-ACL injury were requested and subsequently provided for four studies [28, 32, 34, 36]. Of these four, one study [28] was the only report assessing the included risk factors of kinematics and kinetics, and therefore, although data were made available, it was not possible to include these data in any meta-analysis. Consequently, this study was not part of further analysis or descriptive results. Three articles [42–44] reported partly on the same individuals. Of these, we included the article that provided most data on risk factors and sufficient statistics [42]. Data from three other articles [45–47] were also included in previously published studies [48–50]. Of those, the articles with the greatest sample sizes [45, 47, 49] were included. Consequently, 44 articles were included in this review [8, 10, 32, 34, 36, 42, 45, 47, 49, 51–85] and were assessed for methodological quality (Fig. 1).

### Study Characteristics

Of the 44 included studies, nine [53, 57, 59, 61, 67, 68, 70, 85] were excluded from the meta-analysis due to being the only study reporting on a specific factor or using specific statistics that were not possible to calculate to odds ratios. The caracteristics and results of these studies are reported in online resource 2. Hence, 35 studies in total (16 retrospective and 19 prospective), with a follow-up period of 6 months to 20 years, were included in the meta-analysis. Of these, 28 studies reported on sex difference, 15 studies on age at primary injury, three studies on Body Mass Index (BMI), three studies on smoking status, nine studies on family history (defined as any sibling or parent with a history of ACL injury), seven studies on associated injuries, two studies on geometrics, eight studies on type of sport or return to sport and two studies on timing of primary reconstruction as a risk factor for C-ACL injury. No studies on neuromuscular function, such as muscle strength, muscle activation or movement pattern were eligible for meta-analysis according to our criteria. One study included only females [51], information on sex was not available in two studies [77, 81], whereas the remaining studies included both sexes. Twenty-nine studies pooled children and adults, two studies included only adults [10, 32], one study reported separate results for adults and children [75], whereas four studies included only children and adolescents [55, 65, 69, 78] (see Table 1 for characteristics of the individual studies included in the meta-analyses). In addition, fourteen of these included studies also involved potential risk factors (e.g. self-reported function, knee laxity, knee muscle strength and Tanner stage) not eligible for meta-analysis (only study reporting on a specific factor or using specific statistics that were not possible to calculate to odds ratios). These are likewise reported in online resource 2.

**Table 1:** Characteristics of the studies included in the meta-analysis

Article	Study design	Participants (*n*)	Age mean (sd/range)	Activity level /sports participation	Risk factor(s)	Follow-up (mean/range, years)	Number of C-ACL injuries (*n*)	Quality score
Adults and pediatrics								
Allen et al. [51]	Retrospective	180 females	19.6 (6.9)	Tegner score: 7.9 (0.7) at primary injury	Playing soccer, RTS	5.7	19	17/19 = 89%
Annear et al. [36]	Prospective RCT	19 females, 23 males	28.9 (10.6)	NA	Age, sex, associated injuries	10	5	11/19 = 58%
Bourke et al. [52]	Retrospective	241 females, 432 males	29 (13–62)	NA	Age, sex, family history, associated injuries, RTS	16	95	15/19 = 79%
Filbay et al. [32]	Prospective	32 females, 86 males	26 (5)	Moderate-high activity level (non professional) at primary injury	Age, BMI, associated injuries, activity level, smoking status	5	5	17/19 = 89%
Fältström et al. [10]	Retrospective	107 females, 140 males	28.5 (8.2)	Tegner activity level 9 at primary injury	Activity level	NA	66	10/19 = 53%
Fältström et al. [45]	Retrospective	8986 females, 11,838 males	No further injury: 27 (9.9) C-ACL injury: 22.3 (8.4)	Soccer, other contact ball sports, other sport/recreation, other causes (causes of injury)	Age, sex, associated injuries, playing soccer, timing of surgery	0.5–8.6	591	17/19 = 89%
Goshima et al. [54]	Retrospective	160 females, 73 males	21 (14–51)	Tegner score: 7 (0.8) (Time point NA)	Family history	2	29	14/19 = 74%
Grassi et al. [8]	Retrospective	47 females, 147 males	30.7 (10.6)	Pre-operative Tegner score ≥ 7: 44% < 7: 56%	Age, Sex, BMI, smoking, timing of surgery	10	19	15/19 = 79%
Kaeding et al. [56]	Prospective	1123 females, 1365 men	27 (11)	Marx score: 11.3 (5.3) at primary injury	Age, sex, associated injuries, RTS	2	88	15/19 = 79%
Leys et al. [60]	Prospective	85 females, 95 males	24.5 (13–52)	81% participated in moderate-to-streneous activity at primary injury	Age, sex	15	34	15/19 = 79%
Maletis et al. [62]	Retrospective	6277 females, 11,159 males	27.2 (IR: 18.7–37-7)	NA	Age, sex, BMI	2.4	324	15/19 = 79%
Mardani-Kivi et al. [63]	Retrospective	179 females, 836 males	34 (8.9)	Sport inactivity – regular sport activity (Time point NA)	Sex, BMI, family history	6.5	83	14/19 = 74%
McPherson et al. [34]	Prospective	118 females, 211 males	25.3 (8.7)	Sports participation at primary injury	Sex	2	18	15/19 = 79%
Mohtadi et al. [64]	Prospective RCT	147 females, 183 males	28.5 (9.8)	Tegner Score ≥ 5 at primary injury	Sex	2	17	16/19 = 84%
Nakase et al. [66]	Retrospective Case–control	174 females, 50 males	No injury: 19.3 (4.4) C-ACL injury; 17.5 (4)	Tegner score No injury: 7.0 (0.7) C-ACL: 7.2 (0.8) (Time point NA)	Age	NA	24	13/19 = 68%
Paterno et al. [47]	Prospective	59 females, 19 males	17.1 (3.1)	IKDC Level 1–2 at primary injury	Sex	2 (after RTS)	16	13/19 = 68%
Pinczewski et al. [71]	Prospective	85 females, 95 males	25 (13–42)	Pivoting, cutting or side-stepping sports at primary injury	Sex	10	29	14/19 = 74%
Pujol et al. [72]	Prospective	53 females, 52 males	Females: 17 (2) Males: 18 (2.1)	Alpine Skiers at primary injury	Sex	26	23	14/19 = 74%
Rosenstiel et al. [73]	Retrospective	22 females, 48 males	23.2 (15–37)	Tegner score: 9.3 (1) at primary injury	Sex	3.9	10	15/19 = 79%
Salmon et al. [75]	Prospective	74 females, 105 males	25.8	Streneous, moderate or light activity at follow-up	Age, sex, family history	19.7	22	14/19 = 74%
Salmon et al. [76]	Prospective	67 20 Females47 males	27	Pivoting, cutting or side-stepping sports at primary injury	Age, sex	13	15	15/19 = 79%
Salmon et al. [74]	Prospective	289 females, 383 males	28 (14–62)	IKDC level 1–4 at primary injury	Sex, family history, associated injuries, RTS	5	35	15/19 = 79%
Schickendantz et al. [77]	Retrospective	29 unilateral, 19 bilateral (females/males NA)	23.5	NA	Geometrics	NA	24	11/19 = 58%
Shelbourne et al. [80]	Prospective	552 females, 863 males	21.6 (3.6)	Tegner score: > 7 at primary injury	Sex, age, timing of RTS	5	75	14/19 = 74%
Shelbourne et al. [79]	Prospective	234 women, 480 men	24.3	Noyes score: 99.7% > 12 at primary injury	Sex	NA	27	17/1 9 = 89%
Souryal et al. [81]	Retrospective	50 unilateral, 41 C-ACL (females/males NA)	19 (13–38)	89% participated in sports at primary injury	Geometrics	4	41	10/19 = 53%
Sousa et al. [82]	Prospective	131 females, 92 males	22 (12–59)	Tegner score: > 6.5 at primary injury	Timing of RTS	4	17	17/19 = 89%
Thompson et al. [42]	Prospective	44 females, 46 men	25 (15–42)	Pivoting, cutting or side-stepping sports at primary injury	Age, sex, family history	20	27	15/19 = 79%
Wasserstein et al. [83]	Retrospective	4708 females, 8259 males	29.5 (10.5)	NA	Age, sex, associated injuries	5.2	595	16/19 = 84%
Webster et al. [49]	Retrospective	191 females, 370 males	28.5 (9.9)	NA	Age, sex, family history, RTS	4.8	42	16/19 = 84%
Wright et al. [84]	Prospective	110 females, 125 males	24 (11–54)	NA	Sex	2	7	12/19 = 63%
Pediatrics only								
Heath et al. [55]	Prospective	82 females, 166 males	14.6 (8–17.9)	> 86% participated in sports at primary injury	Age, sex, family history	4.5	28	15/19 = 79%
Morgan et al. [65]	Prospective	104 females, 138 males	13–18	> 91% participated in sports at primary injury	Age, sex, family history, RTS	16.5	48	14/19 = 74%
Perkins et al. [69]	Retrospective	197 females, 157 males	15.3 (10–19)	NA	Sex	2	24	15/19 = 79%
Schmale et al. [78]	Retrospective	23 females, 6 males	14 (1.0)	Tegner score: 8 at primary injury	Sex	4	8	13/19 = 68%

*C-ACL* contralateral, *NA* not available, *RTS* return to sport

### Synthesis of Results

Meta-analyses consisting of between 2 and 28 studies, were performed separately for each C-ACL risk factor. The number of included studies for each meta-analysis is presented below.

#### Sex

**Fig. 2:**
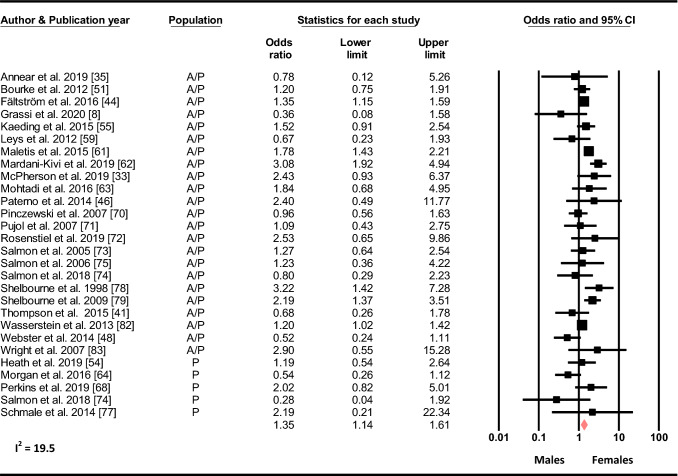
Sex differences in the odds of sustaining a C-ACL injury (C-ACL injury *n* = 2259, controls *n* = 57 189). *A/p* adults and pediatric; *p* pediatric, *NA* not available

Based on 28 studies, females had increased odds of sustaining a C-ACL injury compared to males (OR 1.35, 95% CI 1.14–1.61, *p* < 0.001, C-ACL injury *n* = 2 259, controls *n* = 57 189 (Fig. 2).

#### Age

**Fig. 3:**
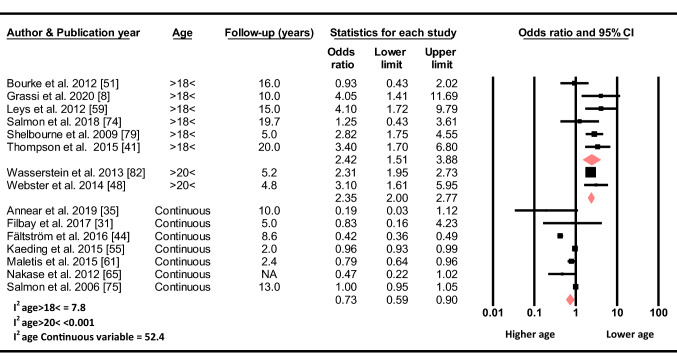
Differences in the odds of sustaining a C-ACL injury between those older and younger than 18 (C-ACL injury *n* = 271, controls *n* = 2 412) and 20 (C-ACL injury *n* = 637, controls *n* = 12 530) years, respectively, and age as a continuous variable (C-ACL injury *n* = 1 052, controls *n* = 38 896). *NA* not available

Based on seven studies, the odds for sustaining a C-ACL injury decreased by 0.27 for every yearly increase in age (OR 0.73, 95% CI 0.59–0.90, *p* = 0.003, C-ACL injury *n* = 1 052, controls *n* = 38 896). Similarly, based on two studies, the odds of sustaining a C-ACL injury was 2.35 times higher for those younger than 20 years compared to those older than 20 years (OR 2.35, 95% CI 2.00–2.77, *p* < 0.001, C-ACL injury *n* = 637, controls *n* = 12 530) and, based on six studies, 2.42 times higher for those younger than 18 years compared to those older than 18 years (OR 2.42, 95% CI 1.51–3.88, *p* < 0.001, C-ACL injury *n* = 271, controls *n* = 2 412) at the time of initial ACL injury (Fig. 3).

#### Body Mass Index

**Fig. 4:**
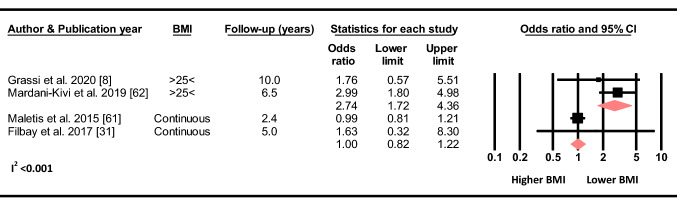
Differences in the odds of sustaining a C-ACL injury between those with a BMI > 25 compared to < 25 (C-ACL injury *n* = 102, controls *n* = 1 080) and BMI as a continuous variable (C-ACL injury *n* = 329, controls *n* = 16 794)

Based on two studies, there was no association between BMI as a continuous variable and the odds of sustaining a C-ACL injury (OR 1.0, 95% CI 0.82–1.22, *p* = 0.996, C-ACL injury *n* = 329, controls *n* = 16 794). In contrast, when dichotomized, a BMI < 25 compared to ≥ 25 was associated with increased odds for subsequent C-ACL injury (OR 2.73, 95% CI 1.73–4.36, *p* < 0.001, C-ACL injury *n* = 102, controls *n* = 1 080) (Fig. 4).

#### Family History

**Fig. 5:**
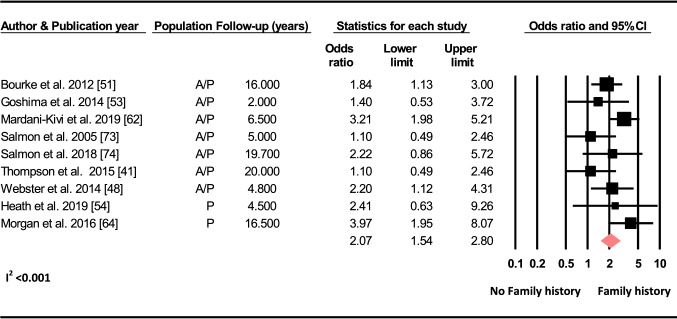
Differences in the odds of sustaining a C-ACL injury between those with a family history of ACL injury and those without (C-ACL injury *n* = 246, controls *n* = 2 590). *A/p* adults and pediatric, *p* pediatric

Based on nine studies, there was a two-fold increase in the odds of sustaining a C-ACL injury with a positive family history of ACL injury (OR 2.07, 95% CI 1.54–2.80, *p* < 0.001, C-ACL injury *n* = 246, controls *n* = 2 590) (Fig. 5).

#### Smoking Status

**Fig. 6:**
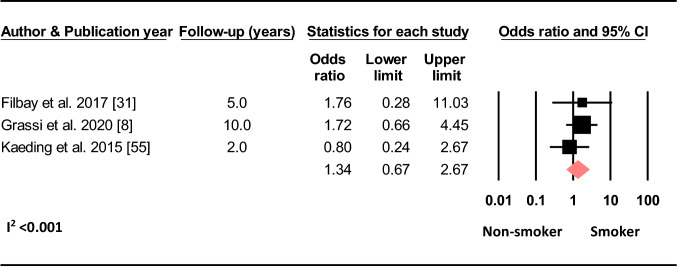
Difference in the odds of sustaining a C-ACL injury between smokers and non-smokers (C-ACL injury *n* = 46, controls *n* = 2 629)

Based on three studies, there was no effect of being a smoker compared to being a non-smoker on the odds of sustaining a C-ACL injury (OR 1.34, 95% CI 0.67–2.67, *p* = 0.411, C-ACL injury *n* = 46, controls *n* = 2 629) (Fig. 6).

#### Geometrics

**Fig. 7:**
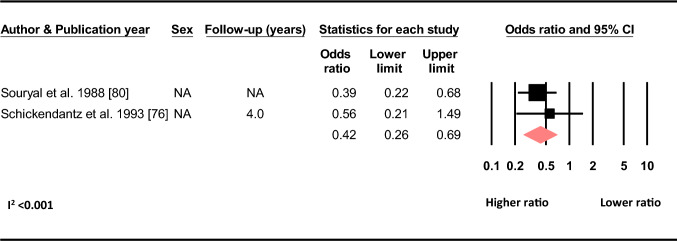
Differences in the odds of sustaining a C-ACL injury with increasing width of the intercondylar notch/width of the distal femur ratio (C-ACL injury *n* = 84, controls *n* = 1 319). *NA* not available

Based on two studies, there was a decrease of 0.58 in the odds of sustaining a C-ACL injury with an increase in the ratio of the width of the intercondylar notch and the width of the distal femur (OR 0.42, 95% CI 0.26–0.69, C-ACL injury *n* = 84, controls *n* = 1 319) (Fig. 7).

#### Associated Injuries to the Ipsilateral Knee

**Fig. 8:**
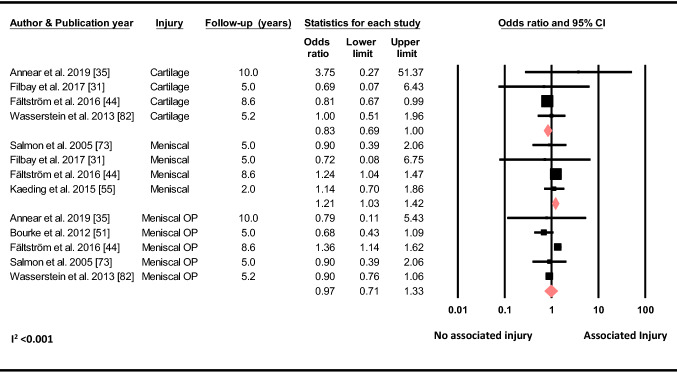
Differences in the odds of sustaining a C-ACL injury between those with and without concomitant cartilage injury (C-ACL injury *n* = 1198, controls *n* = 31,708), meniscal injury (C-ACL injury *n* = 719, controls *n* = 22 475) and meniscal surgery (C-ACL injury *n* = 1321, controls *n* = 32 738). *Op* surgery

Based on four studies, the meta-analysis showed that having a concomitant meniscal injury increased the odds of a C-ACL injury (OR 1.21, 95% CI 1.03–1.42, *p* = 0.020, C-ACL injury *n* = 719, controls *n* = 22 475), whereas, based on 5 studies, having meniscal surgery (OR 0.97, 95% CI 0.71–1.33, *p* = 0.859, C-ACL injury *n* = 1 321, controls *n* = 32 738) did not. A meta-analysis of four studies showed that concomitant cartilage injury decreased the odds of sustaining a C-ACL injury (OR 0.83, 95% CI 0.69–1.00, *p* = 0.050, C-ACL injury *n* = 1 198, controls *n* = 31 708) (Fig. 8).

#### Timing of Reconstruction

**Fig. 9:**
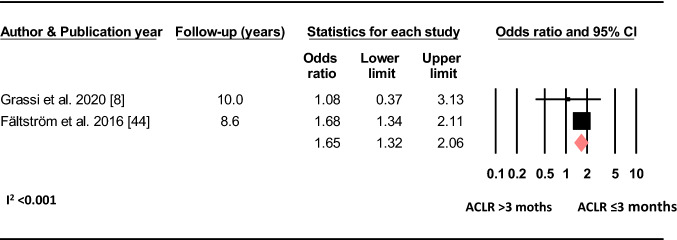
Differences in the odds of sustaining a C-ACL injury between those who performed the reconstruction > 3 months and those who performed the reconstruction ≤ 3 months post primary injury (C-ACL injury *n* = 571, controls *n* = 17 842). *ACLR* anterior cruciate ligament reconstruction

Based on two studies, receiving reconstruction of the primary ACL injury ≤ 3 months post injury increased the odds of sustaining a C-ACL injury (OR 1.65, 95% CI 1.32–2.06, *p* < 0.001, C-ACL injury *n* = 571, controls *n* = 17 842) (Fig. 9).

#### Activity Level and Sports Participation

**Fig. 10:**
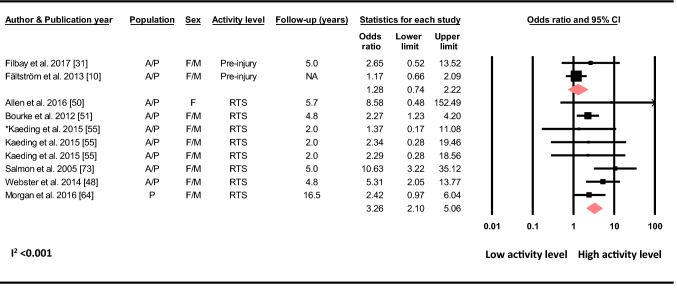
Differences in the odds of sustaining a C-ACL injury according to pre-primary injury activity level (Pre-injury) (C-ACL injury *n* = 71, controls *n* = 291) and between those who returned to a high activity level/sport (RTS) and those who did not (C-ACL injury *n* = 327, controls *n* = 4256). *A/p* adults and pediatric, *F* females, *M* males, *NA* not available. “Asterisk” Kaeding et al. reported for three different cohorts, returning to basketball, football and soccer, respectively

Based on two studies, Tegner activity level prior to the initial injury was not associated with the odds of sustaining a C-ACL injury (OR 1.29, 95% CI 0.74–2.22, *p* = 0.368, C-ACL injury *n* = 71, controls *n* = 291) (Fig. 10).

Based on six studies, returning to a high activity level/sport (sports including cutting and pivoting) increased the odds of sustaining a C-ACL more than threefold compared to returning to a low activity level or not returning at all (OR 3.26, 95% CI 2.10–5.06, *p* < 0.001, C-ACL injury *n* = 327, controls *n* = 4 256) (Fig. 10).

**Fig. 11:**
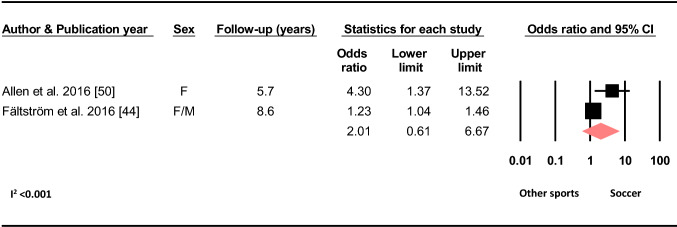
Difference in the odds of sustaining a C-ACL injury between those who played soccer at the time of primary injury and those who played other sports (C-ACL injury *n* = 578, controls *n* = 17 599). *F* females, *M* males

Based on two studies, playing soccer at the time of primary injury did not increase the odds of sustaining a C-ACL compared to other sports (OR 2.01, 95% CI 0.61–6.67, *p* = 0.252, C-ACL injury *n* = 578, controls *n* = 17 599) (Fig. 11).

**Fig. 12:**
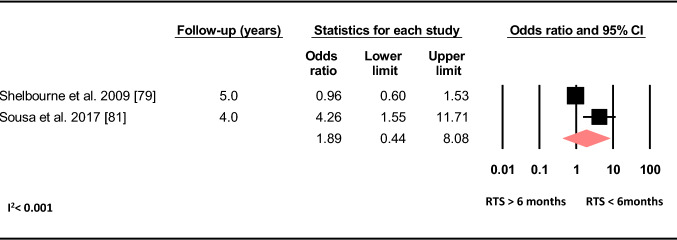
Difference in the odds of sustaining a C-ACL injury between those who returned to sport > 6 months post ACLR and those who returned to sport < 6 months post ACLR (C-ACL injury *n* = 92, controls *n* = 1 475). *ACLR* anterior cruciate ligament reconstruction, *RTS* return to sport

A meta-analysis of two studies showed no increased odds of sustaining a C-ACL with returning to sport ≤ 6 months post primary ACLR compared to more than 6 months (OR 1.89, 95% CI 0.44–8.08, *p* = 0.392, C-ACL injury *n* = 92, controls *n* = 1 475) (Fig. 12).

#### Subgroup Analysis of Pediatric Populations

##### Sex

Based on five studies, there was no sex difference in the risk of sustaining a C-ACL (OR 0.97, 95% CI 0.50–1.89, *p* = 0.937, C-ACL injury *n* = 130, controls *n* = 754) (Online resource 3, Fig. 1).

##### Age

Based on two studies, there was no difference in the risk of sustaining C-ACL between those younger than 14 years compared to those 14–18 years old (OR 0.77, 95% CI 0.23–2.64, *p* = 0.688, C-ACL injury *n* = 78, controls *n* = 337) (Online resource 3, Fig. 2).

##### Family History

Based on two studies, there was an increased odds of sustaining a C-ACL with a family history of ACL injury (OR 3.56, 95% CI 1.90–6.67, *p* < 0.001, C-ACL injury *n* = 78, controls *n* = 337) (Online resource 3, Fig. 3).

### Sensitivity Analysis

The sensitivity analyses revealed that excluding the studies that only included children or adolescents (sex, family history, return to high activity level) did not change the direction or significance of the results. Limiting the studies to those with a follow-up ≥ 2 years did not change the direction or significance of the results for sex, family history, meniscal injury or return to a high activity level, whereas lower age at primary injury (continuous varable) did no longer increase the odds of sustaining a C-ACL injury (Online resource 4).

### Heterogeneity and Risk of Bias

I^2^ ranged between < 0.001% and 19.5% for all meta-analyses except one (age as a continuous variable), indicating low heterogeneity between studies [86]. The analysis for age as a continuous variable, however, had an I^2^ of 52.4%, indicating moderate heterogeneity [86].

The quality of the included studies ranged from 53 to 95% with a mean score of 77.3%, indicating a generally high methodological quality (Online resource 5, Table 1). Sex was the only variable for which more than ten articles were included in the meta-analysis and a funnel plot with trim and fill imputations was eligible. The funnel plot showed no difference in effect size if the apparent biases were removed, indicating no publication bias for sex as a risk factor for C-ACL [25] (Online resource 5, Fig. 1).

## Discussion

In this systematic review with meta-analysis, we found that female sex, younger age (< 18), BMI < 25, a family history of ACL injury, femoral geometrics, concomitant meniscal injury, reconstruction of primary ACL injury perfomed within 3 months after injury, and returning to a high activity level (highest odds), were all independently associated with increased odds of sustaining a subsequent C-ACL injury. The analysis revealed no influence of smoking status, pre-injury activity level, playing soccer compared to other sports or timing of return to sport on the odds for sustaining a C-ACL injury. Few studies were identified that investigated sensorimotor and neuromuscular factors, such as proprioception, muscle strength, muscle activation patterns and/or kinematics and kinetics as risk factors for C-ACL injury and meta-analyses were, therefore, not possible.

Females have previously been reported to have about three times higher risk of sustaining a primary ACL injury compared to males [1, 87–90]. The current meta-analysis, including 59 448 individuals, suggests females to have 35% higher odds (OR 1.35) of sustaining a subsequent C-ACL injury compared to their male counterparts, which is a substantially lower risk compared to that of primary injury. Several hormonal, neuromuscular and biomechanical risk factors have been proposed to contribute to the apparent sex difference in primary ACL injury rate. For example, the menstrual cycle and the use of contraceptives have been associated with ACL injury risk [91]. Furthermore, females seem to exhibit greater knee joint laxity, altered hip and knee muscle activation patterns as well as decreased neuromuscular control of the trunk and hip compared to males, which may contribute to a greater injury risk [16]. Females both with and without ACL injuries also perform functional tasks with greater knee valgus, a movement pattern commonly associated with knee injury risk [92], compared to males [23, 93]. On the other hand, the sub-group analysis for studies including only children and/or adolescents showed no sex difference for the risk of sustaining a C-ACL injury in individuals younger than 19 years. This result may be explained by the fact that the aforementioned differences in biomechanics and neuromuscular function seem to develop through maturation. Shultz et al. [94]., reported that while females and males presented with similar anatomical features in early maturation stages, females developed more valgus aligned knee postures and increased knee laxity during growth, whereas males moved towards a varus aligned position and decreased knee laxity. That the C-ACL injury risk for females compared to males seems to be much less compared to primary injury may potentially be explained by the fact that some of the risk factors for primary injury may not be relevant for C-ACL injury, in addition to that some risk factors for primary injury, such as family history [54] and kinetic and postural stability deficits [30] are present in both males and females who sustain the first injury. As no studies which included hormonal cyclic variations, knee joint laxity or neuromuscular factors, such as muscle strength and musle activation patterns were eligible for meta-analysis in the current review, it cannot be ruled out that such sex-specific risk factors for primary injury may also contribute to an increased risk of subsequent C-ACL injuries in adult females.

The result from the present meta-analysis supports the findings of a previous review that reported an increased rate of secondary ACL injuries (ipsi-lateral and contra-lateral injuries combined) in individuals younger than 25 years [9]. Similarly, we found the odds of sustaining a C-ACL injury in those younger than 18 and 20 years to be twice the odds of those older than 18 and 20 years, respectively.There are major development regarding anatomical, biomechanical and neuromuscular features as well as changes in joint laxity still during the adolescent period, which may contribute to the risk of injury in this age [94, 95]. The fact that we found no age difference (< 14 vs. > 14–18 years) in the odds of sustaining a C-ACL injury in a group consisting of only children and adolescents may support this theory. In the meta-analysis, returning to a high activity level was associated with a threefold increase in the odds of sustaining a C-ACL injury. Another explanation for the association between younger age and injury risk may be that younger individuals tend to return earlier, more often and to a higher activity level compared to their older peers [40, 49, 80].

To have a family history of ACL injury was associated with a twofold increase in the odds of sustaining a C-ACL injury in the pooled analysis for adults and younger individuals, and with a threefold increase in individuals younger than 18 years. The influence of genes and associated polymorphisms has previously been suggested to play a role for knee injury [96]. Results from different studies are, however, conflicting and there is currently no convincing evidence that specific genotypes will pre-dispose individuals to ACL injury [96–100]. Genetic variations in the form of inherited anatomical or biomechanical alterations may, however, be important for injury risk. Salmon et al. [74] reported a Hazard ratio of 7.3 to sustain a C-ACL injury in those with a tibial slope of ≥ 12° compared to those with a tibial slope < 12° (Online resource 2). A recent meta-analysis found that individuals with ACL injury had smaller femoral notch width and lower notch width index compared to non-injured individuals [101]. Our meta-analysis also revealed that individuals with a higher width of the intercondylar notch/width of the distal femur ratio had lower odds of sustaining a C-ACL injury. Another explanation for the relation between family history of ACL injury and the risk of C-ACL injury may be related to an inherited culture of sports participation. In a study by Goshima et al. [54], all family members of the study participants that had a history of ACL injury were injured during sports and 65% were injured during the same sport as the their relative included in the study. Taken together, it is possible that the explanation for the association between family history and C-ACL injury is partly attributed to genetic variations in the morphology of the knee and/or a mutual familial interest for sport participation. Future studies are, however, warranted to confirm this theory and to investigate possible associations between genetic variations in other physical characteristics and the risk of secondary injury.

In the current meta-analysis we found an increased odds of a C-ACL injury if the participant had a concomitant meniscal injury to the ipsilateral knee at the time of primary injury, whereas no such association was revealed if a meniscal surgery was performed with the primary reconstruction. Interestingly, individuals that had concomitant cartilage injury on the index knee had lower odds of sustaining a subsequent C-ACL injury. It may be speculated that individuals with severe meniscal damage which requires surgery and those with cartilage injury may not return to sport to the same extent and, thus, are less likely to put their knee at risk. However, given the low ORs (≤ 1.21) and contradicting results, the clinical relevance of associated meniscal and cartilage injuries for further C-ACL injuries has still to be determined. Furthermore, a delay in surgery as well as a higher BMI have previously been linked to developing associated meniscal and cartilage injuries in individuals undergoing ACL reconstruction [102–104]. In the current meta-analysis, an early reconstruction (< 3 months vs. ≥ 3 months) and a lower BMI (< 25 vs. ≥ 25) increased the odds of sustaining a C-ACL injury. Likewise, it is possible that individuals that perform an early surgery and/or have a lower BMI, are more likely to participate in sport and may also return earlier to sport than those with a delayed surgery and/or higher BMI and thereby increase the risk of a new injury. However, these assumptions need to be corroborated by further research.

Participation in high-risk sports which include cutting and pivoting movements, such as soccer, basketball and handball, is widely accepted to substantially increase the risk of ACL injuries [105]. In the current review, playing soccer compared to other sports at the time of primary injury did not increase the risk of subsequent C-ACL injury, whereas returning to a high activity level, irrespective of sport type, was associated with the highest odds (3.26) of sustaining a C-ACL injury. This indicates that returning to any knee-demanding sport will put the athlete at greater risk for subsequent injuries. However, in the articles on playing soccer compared to other sports, other sports were not clearly defined. If other sports also included sports involving cutting and pivoting, this may be an explanation for the lack of difference in injury risk between soccer and other sports. On the other hand, activity level prior to the primary injury was not associated with the risk for C-ACL injury. It should be noted that in the two studies that were included in the meta-analysis for pre-injury activity level, one reported a median Tegner activity level of 9 [10] and one reported approximately 80% of the participants to have a pre-injury Tegner of 8–9 [32]. This suggests that most participants in these studies participated in high-risk sports and consequently the narrow distribution may have influenced the result for this analysis.

There is conflicting evidence whether an early return to sport may increase the risk of subsequent ACL injuries (graft rupture or graft rupture and C-ACL injury combined) [106–108]. In the current review, timing of return to sport (< 6 months vs. ≥ 6 months) did not affect the C-ACL injury rate. No studies were identified that investigated other time points of return to sports in relation to C-ACL injury specifically. It has been suggested that the risk for further injuries may not be explained by time alone but may rather be related to the individuals’ functional capacity at the time of return to sport [109, 110]. There is, however no clear evidence supporting that adequate functional capacity (i.e., passing certain return to sport criteria) will decrease the risk of re-injury [109, 110]. A recent meta-analysis reported that passing such criteria may even increase the risk of secondary C-ACL injury [110]. Future studies are, thus, warranted in order to clarify the influence of neuromuscular function as well as different time points of return to sport on secondary ACL injuries.

Smoking status was not related to the risk of subsequent C-ACL injury in this review. This is in line with studies showing no effect of smoking status and the risk of revision surgery after ACLR [111, 112]. Although smokers seem to have worse self-reported and clinical outcomes and increased risk of complications after ACLR [112], being a smoker seems not to influence the risk of further injury to either knee.

Most studies included in this review investigated risk factors related to demographics and/or sports participation, while studies on elements related to neuromuscularfunction as possible risk factors for sustaining a C-ACL injury are lacking. A few studies have previously pointed to a possible role of hip and knee movement patterns and moments during functional tasks, as well as hop performance, for the risk of sustaining second ACL injuries (graft rupture and C-ACL injury combined) [30, 39, 113]. There is nevertheless limited evidence for lower extremity strength as a contributing factor to C-ACL injury risk [53, 66] (Online resource 2). Given that demographic factors such as sex, age and family history cannot be changed, studies on risk factors that are modifiable are encouraged. The possibility of identifying factors that are modifiable by training will facilitate the design of rehabilitation protocols to better reduce the risk of secondary injuries following ACL injury.

A strength of this review is that we considered all studies for inclusion without restrictions relating to sex, age, sports participation or publication date, indicating high generalizability of our results. Additionally, the studies included were in general of high methodological quality and the separate meta-analyses included between 360 and 59,000 individuals. This review does nonetheless have some limitations. While we included all studies that reported C-ACL injuries, some studies used subsequent C-ACL reconstruction (mainly identified from surgical records) as their primary outcome, whereas some studies used subsequent C-ACL injury reported by the participants or by medical staff. It is likely that the use of C-ACL reconstruction as primary outcome underestimated the incidence of further knee injuries, which may have influenced the results in these included studies. Secondly, we pooled studies with different follow-ups (6 months to 20 years) in the analyses. Several studies show that the time from primary reconstruction to subsequent C-ACL injury is often 3–4 years [8, 52, 73], indicating that studies with a follow-up of less than 3 years may not be able to capture all C-ACL injuries and consequently the results from such studies should be interpreted with caution. Our sensitivity analysis also showed that lower age (as a continuous variable) was no longer a risk factor for C-ACL injury if studies with a follow-up of ≤ 2 years were excluded, whereas there were no differences in the result for other risk factors. Thirdly, to increase power, we pooled studies that included adults only, children only and those who pooled children and adults. It may be argued that the risk factors for children and adults are not the same. We did, however, perform both a sensitivity analysis, excluding studies on children and subgroup analysis including children only to account for these possible differences. Forth, two of the meta-analysis included only two studies each with a relatively low number of individuals with C-ACL injury, i.e., the analysis for pre-injury activity level (*n* = 71) and the analysis for timing of return to sport (*n* = 92). It has been suggested that there will be an increased risk of overestimating the effect when meta-analysis with a low number of events are performed [114]. Thus, some caution is needed when interpreting the result for pre-injury activity level and timing of return to sport. Fifth, although addressing relevant risk factors, a few articles could not be included in the meta-analyses due to being the only study assessing a specific variable or time point (e.g., different geometric variables and muscle strength pre/post reconstruction). To fully understand the role of potential risk factors for C-ACL injury, a more standardized methodological approach to the assessment of such factors should be considered in future studies. Finally, the risk of sustaining a C-ACL injury is multifactorial in nature and therefore cannot be explained simply by any single risk factor, and many of these are also interrelated. For example, it has been suggested that younger age is related to an earlier return to sport and higher activity level [40] as well as reflecting neuromuscular and anatomical characteristcs [94, 95]. Likewise, the analysis for sex difference may be influenced by associated genetic, anatomical and neuromuscular differences between the sexes [94]. In the current review we performed separate analyses for each risk factor. Meta-regressions, adjusting for confounding factors were not part of the aim of this study and indeed not even possible, due to limited availability of data and a limited number of studies in each analysis [115]. It is thus possible that the results for some of the factors that we found to be significant predictors for C-ACL injury in the meta-analyses would have been different if other variables had been poosible to take into account by applying a multifactorial model.

## Conclusion

The results from this systematic review with meta-analysis including up to ≈59,000 individuals reveal that return to a high activity level was the most prominent risk factor for sustaining a contra-lateral secondary ACL injury. Other independently associated factors were female sex, younger age and family history of ACL injury. All of these factors should be considered when screening for individuals that are at high risk of sustaining a C-ACL injury. Since most studies included in this review investigated demographic factors which are non-modifiable in nature, future studies are encouraged to investigate the contributing role of neuromuscular factors, such as muscle strength, muscle activation and movement patterns, that can be modified by training in order to target interventions which may better reduce secondary ACL injury risk.

## Supplementary Information

Below is the link to the electronic supplementary material.

Supplementary file 1 (DOCX 48 KB)

Supplementary file 2 (DOCX 45 KB)

Supplementary file 3 (DOCX 60 KB)

Supplementary file 4 (DOCX 39 KB)

Supplementary file 5 (DOCX 112 KB)

## Declarations

### Availability of Data and Material

The datasets used and/or analyzed during the current study are available from the corresponding author on reasonable request.
