# Supplementary material for: Risk Factors for Contra-Lateral Secondary Anterior Cruciate Ligament Injury: A Systematic Review with Meta-Analysis
Source: Sports Med. 2021 Jan 30;51(7):1419–38. doi: 10.1007/s40279-020-01424-3 (PMC8222029; doi:10.1007/s40279-020-01424-3)
Supplement: Supplementary file 1 — (DOCX 48 KB) [file 40279_2020_1424_MOESM1_ESM.docx]

**Online resource 1.** Quality index checklist adapted from Downs and Black

| **ITEM** | | **Yes** | **Unable to determine** | **No** | **N/A** | **Comment** |
| --- | --- | --- | --- | --- | --- | --- |
| 1. | Is the hypothesis/aim/objective of the study clearly described? | □ |  | □ |  |  |
| 2. | Are the main outcomes to be measured clearly described in the Introduction or Methods sections? | □ |  | □ |  |  |
| 3. | Are the characteristics of the subjects included in the study clearly described? | □ |  | □ |  |  |
| 5. | Are the distributions of principle confounders in each group of subjects to be compared clearly described? | □ | □  partially | □ |  |  |
| 6. | Are the main findings of the study clearly described? | □ |  | □ |  |  |
| 7. | Does the study provide estimates of the random variability in the data for the main outcome? | □ |  | □ |  |  |
| 10. | Have actual probability values been reported (e.g. 0.035 rather than <0.05) for the main outcomes except where the probability value is less than 0.01? | □ |  | □ |  |  |
| *External validity* | |  |  |  |  |  |
| 11. | Were the subjects asked to participate in the study representative to the entire population from which they were recruited? | □ | □ | □ |  |  |
| 12. | Were those subjects who were prepared to participate representative of the entire population from which they were recruited? | □ | □ | □ |  |  |
| *Internal validity – Bias* | |  |  |  |  |  |
| 15. | Was an attempt made to blind those measuring the main outcome? | □ | □ | □ |  |  |
| 16. | If any of the results were based on “data dredging“, was this made clear? | □ | □ | □ |  |  |
| 18. | Were the statistical tests used to assess the main outcomes appropriate? | □ | □ | □ |  |  |
| 20. | Were the main outcome measures used accurate (valid and reliable)? | □ | □  Accuracy not reported but method clearly described | □ |  |  |
| *Internal validity – confounding (selection bias)* | |  |  |  |  |  |
| 21. | Were the subjects (e.g. the two groups to be compared) recruited from the same population? | □ | □ | □ |  |  |
| 22. | Were the study subjects (the two groups to be compared) recruited over the same period of time? | □ | □ | □ |  |  |
| 25. | Were there adequate adjustments for confounding in the analyses from which the main findings were drawn? | □ | □ | □ |  |  |
| *Bias* | |  |  |  |  |  |
| 27. | Did the study have sufficient power to detect a clinically important effect? | □ | □ | □ |  |  |

Every question was given 1 point for ”yes” and zero points for ”unable to determine” and ”no” except for item 5 and 20, where 2 points were given for “yes” and 1 point for “partially” and “Accuracy not reported but method clearly described”. To be able to receive 2 points for item 20, the studies had to report accuracy for all included outcomes.
