# Supplementary material for: Risk Factors for Contra-Lateral Secondary Anterior Cruciate Ligament Injury: A Systematic Review with Meta-Analysis
Source: Sports Med. 2021 Jan 30;51(7):1419–38. doi: 10.1007/s40279-020-01424-3 (PMC8222029; doi:10.1007/s40279-020-01424-3)
Supplement: Supplementary file 2 — (DOCX 45 KB) [file 40279_2020_1424_MOESM2_ESM.docx]

**Online resource 2** Characteristics and results of the articles and factors excluded from the meta-analysis

| **Article** | **Study design** | | **Participants**  (n) | **Age**  Mean (SD/range) | **Activity level/sports participation**  Mean (SD) | **Risk factor(s)** | **Follow-up**  (mean/range, years) | **Number of C-ACL injuries**  (n) | **Primary reason for exclusion from meta-analysis** | **Results** | **Quality score** |
| --- | --- | --- | --- | --- | --- | --- | --- | --- | --- | --- | --- |
|  | | *Articles not included in the meta-analysis* | | | | | | | | | |
| Davey et al. 2019 | Prospective | | 55 females | 17.1 (2.14) | Hours per week sports participation: 9.35 (6.37) at primary injury | Age, geometrics, anterior knee stiffness, sport participation, trunk, hip, knee and foot strength, BMI, lower extremity alignment, general laxity, knee joint laxity, personality (pre-reconstruction) | 3.8 | 11 | Statistics not possible to re-calculate to OR | Decrease in knee stiffness (HR = 2.37, p = 0.042), lower age (HR = 1.70, p = 0.004), fewer hours of sports participation (HR = 1.19, p = 0.048), increase in hip anterversion (HR = 2.38, p = 0.017) associated with C-ACL injury. No other associations (p > 0.05) | 17/19 = 89% |
| Lai et al. 2018 | Prospective | | 158 (females/males NR | 23.5 (3.6) | AFL players at primary injury | Age, family history | 2-18 | 20 | Statistics not possible to re-calculate to OR | < 21 years at the time of primary injury (RR: 3.2, p = 0.004) and a positive family history of ACL injury (RR: 3.8, p = 0.02) associated with C-ACL | 13/19 = 68% |
| Larson et al. 2017 | Prospective | | 102 females, 81 males | 26.9 (12-62) | NA | General Laxity | 6 | 10 | Only article reporting on specific risk factor | No difference in general laxity between those who sustained a C-ACL injury and those who did not (9.8% vs 4.2 %, p = 0.235) | 14/19 = 74% |
| Levins et al. 2017 | Prospective | | 55 females | 17.5 (2.5) | Sport participation High school/college level at primary injury | Age, geometrics | 3 | 9 | Statistics not possible to re-calculate to OR | Increased risk of C-ACL injury with decrease in age (HR = 0.53, p = 0.05), femoral inter condylar notch width (HR = 1.88, p = 0.016), medial-lateral width of lateral tibial spine (HR = 3.59, p = 0.039,  height of medial tibial spine (HR = 1.75, p = 0.045 and thickness of the articular cartilage posterior region of medial tibial compartment (HR = 2.15, p = 0.014). No associations between 31 other tibial and femoral geometric measures (p > 0.05) | 17/19 = 89% |
| Magnussen et al. 2018 | Prospective | | 1023 females, 1302 males | 26.7 (11.1) | Recreational/  competetiv sports participation at primary injury | Knee laxity | 6 | 156 | Only article reporting on specific risk factor | Increased odds of sustaining an C-ACL injury with increased pre-operative lachman’s test (OR: 1.68, p = 0.019). No association between pre-operative pivot shift or anterior drawer test (OR: 1.3, p = 0.16 and OR: 1.14, p = 0.64) and C-ACL injury | 18/19 = 95% |
| Patel et al. 2019 | Retrospective | | 521 females, 535 males | 15.1 (2.4) | 92% participated in sports at primary injury | BMI | 2.2 | 45 | Only article defining BMI as percentiles | No difference in the rate of C-ACL injury between those with normal BMI (7.3%) and those with overweight (4.2% ), p = 0.10 | 18/19 = 95% |
| Paterno et al. 2018 | Prospective | | 40 (females/males NA) | 16.2 (3.4) | IKDC level 1-2 at primary injury | Fear of movement/re-injury | 1 (after RTS) | 7 | Only article reporting on specific risk factor | No difference in the Tampa Scale for Kinesiophobia at return to sport after primary ACLR between those who sustained a C-ACL injury, (16.0 (1.8)) and those who did not (mean: 17.3 (4.2)), p = 0.430 | 13/19 = 68% |
| Pfeiffer et al. 2018 | Retrospective | | 38 females, 47 males | 29.1 (9.7) | NA | Geometrics | 4.4 | 35 | Only article reporting on specific risk factor | Those who sustained a C-ACL injury had higher lateral femoral condyle ratio (66.9 (4.3)) compared to those who did not (64.2 (3.8)), p = 0.008 | 16/19 = 84% |
| Webb et al. 2013 | Prospective | | 90 females, 91 males | 26 | NA | Geometrics | 15 | 15 | Only article reporting on specific risk factor | No difference in posterior tibial slope between those who sustained a C-ACL injury (9.9 (2.3)), and those who did not (8.5 (2.2)), p = 0.16 | 16/19 = 84% |
|  | | *Additional excluded risk factors from articles included in the meta-analysis* | | | | | | | | | |
| Fältström et al. 2016 | Retrospective | | 8986 females,  11838 males | No further injury: 29 (9.9)  C-ACL injury: 22.3 (8.4) | Soccer, other contact ball sports, other sport/recreation, other causes (causes of injury) | Self-reported function (KOOS, EQ-5D), | 0.5-8.6 | 591 | Only article reporting on specific risk factor | increased risk of C-ACL injury with better pre-operative KOOS scores of the subscales; Pain (HR = 1.01, p = 0.021), activity of daily living (HR = 1.01, p = 0.003), sport/function (HR = 1.01, p = 0.002) and quality of life (HR = 1.01, p = 0.011). No increased risk associated with pre-operative KOOS symptom score (HR = 1.01,) EQ-5D index (HR = 1.08) or EQ-5D VAS score (HR = 1.01), p > 0.05. Higher risk of C- | 17/19 = 89% |
| Grassi et al. 2020 | Retrospective | | 47 females, 147 males | 30.7 (10.6) | Tegner score  ≥ 7: 44%  < 7: 56% at primary injury | Pre-operative Tegner activity level | 10 | 19 | Only article defining Tegner activity level as >7< | Increased risk of C-ACL injury with a pre-operative activity level of ≥ 7 compared to < 7 (HR = 15.2, p = 0.009) | 15/19 = 79% |
| Heath et al. 2019 | Prospective | | 82 females.  166 males | 14.6 (8-17.9) | > 86% participated in sports at primary injury | Tanner stage, growth plate status | 4.5 | 28 | Only article reporting on specific risk factor | No associations between Tanner stage or growth plate status and the risk of C-ACL (p > 0.78) | 15/19 = 79% |
| Kaeding et al. 2015 | Prospective | | 1184 females,  1498 men | 27 (11) | Marx score: 11.3 (5.3) at primary injury | Activity level at the time of primary reconstruction | 2 | 88 | Only article reporting on specific risk factor and specific time point | increased odds of sustaining a C-ACL with increased Marx activity score at the time of primary reconstruction (OR: 1.12, p < 0.01) | 15/19 = 79% |
| Mardani-Kivi et al. 2019 | Retrospective | | 179 females, 836 males | 34 (8.9) | Sport inactivity – regular sport activity (Time point NA) | Age, BMI, sports participation | 6.5 | 83 | Only article including this specific cut-offs for age, BMI and sports participation | Patients < 30 years, (p = 0.006), BMI 20-25, (p < 0.001), participating in regular sports activities, (p = 0.026) associated with an increased risk of C-ACL injury | 14/19 = 74% |
| McPherson et al. 2019 | Prospective | | 118 females,  211 males | 25.3 (8.7) | Sports participation at primary injury | Timing of RTS | 1 | 18 | Only article reporting on specific risk factor (mean days) | No difference in timing of RTS between those who sustained a C-ACL injury (427.5 (121)) days and those who did not (409 (180)) days, p = 0.61 | 15/19 = 79% |
| Mohtadi et.al 2016 | Prospective RCT | | 147 females, 183 males | 28.5 (9.8) | Tegner Score ≥ 5 at primary injury | Age | 2 | 17 | Only article reporting on specific risk factor (>27< years) | No association in C-ACL injury rate between those age < 27 years (7.1%) compared to those > 27 years (3.1%), p = 0.136 | 16/19 = 84% |
| Nakase et al. 2012 | Retrospective | | 174 females  50 males | No injury: 19.3 (4.4) C-ACL injury; 17.5 (4) | Tegner score No injury: 7.0 (0.7)  C-ACL: 7.2 (0.8) (Time point NA) | Knee laxity at RTS (KT-1000), Tegner (time point NA), Knee strength 6 months post ACLR | NA | 24 | Only article reporting on specific risk factors at specific time point (6 months post ACLR) | No association between Tegner activity score, (OR = 0.66, p = 0.192), Knee laxity at RTS (OR = 0.84, p = 386), knee extension strength (OR = 0.99, p = 0.801), knee flexion strength (OR = 0.99, p = 908) or H/Q-ratio (OR = 1.63, p = 0.923) and C-ACL injury | 13/19 = 68% |
| Pincewski et al. 2007 | Prospective | | 85 females  95 males | 25 (15-42) | Pivoting, cutting or side-stepping sports at primary injury | Age, knee laxity and, activity level at 2 years post ACLR | 10 | 29 | Only article reporting on age >21< years and laxity and activity level at 2 years post ACLR | Increased risk of C-ACL injury in those < 21 years (p = 0.02). No association between knee laxity (anterior drawer, Lachman’s, pivot shift, KT-1000) (p = 0.630) or activity level at 2 years post ACLR and the risk of C-ACL (p = 0.190) | 14/19 = 74% |
| Rosenstiel et al. 2019 | Retrospective | | 22 females,  48 males | 23.2 (15-37) | Tegner score: 9.3 (1) at primary injury | Age, associated injuries, pre-op knee laxity, contact vs non-contact sports | 3.9 | 10 | Statistics not possible to re-calculate to OR | No association between age < 20 years compared to age > 20 years (HR = 0.42, p = 0.201), concomitant medial meniscus injury (HR = 2.11, p = 0.848), lateral meniscus injury (HR = 1.08, p = 0.848, preoperative IKDC grade (arthrometer) (> 10 vs. < 5 degrees, HR = 0.205, p = 0.745), participation in contact sports at the time of primary injury compared to non-contact sports (HR = 1.35, p = 0.750) and the risk of C-ACL. | 15/19 = 79% |
| Salmon et al. 2005 | Prospective | | 289 females,  383 males | 28 (14-62) | IKDC level 1-4 at primary injury | Contact vs. non-contact injury mechanism | 5 | 35 | Only article reporting on specific risk factor | No increased risk of C-ACL injury with contact vs non-contact mechanism, of primary injury (OR = 1.9 p = 0.14) | 15/19 = 79% |
| Salmon et al. 2018 | Prospective | | 74 females,  105 males | 25.8 | Strenuous, moderate or light activity at follow-up | Geometrics | 19.7 | 22 | Only article reporting on specific risk factor with this specific cut-off (>12< degrees) | Increased risk of sustaining a C-ACL injury with a posterior tibial slope ≥ 12 degrees compared to < 12 degrees (HR = 7.3, p = 0.001) | 14/19 = 74% |
| Schikendantz et.al 1993 | Retrospective | | 29 unilateral, 19 bilateral (females/males NA) | 23.5 | NA | Geometrics | NA | 19 | Only article reporting on specific risk factors | No association between 7 different femoral notch and condylar measures and the risk of C-ACL injury, p > 0.148 | 11/19 = 58% |
| Shelbourne et al. 1998 | Prospective | | 234 females,  480 males | 24.3 | Noyes score: 99.7% > 12 at primary injury | Geometrics | NA | 27 | Only article reporting on specific risk factor | Increased rate of C-ACL injury with a femoral inter condylar notch width of < 15 mm (5.9%) compared > 15mm (1.2%), p < 0.01) | 17/19 = 89% |

SD=standard deviation: C-ACL = contra-lateral;, HR = hazard ratio; RR = risk ratio; OR = odds ratio; NR = not reported: BMI = body mass index; ACLR = anterior cruciate ligament reconstruction; KOOS = knee injury and osteoarthritis outcome score; EQ5D = EuroQol – 5-dimension descriptive system; RTS = return to sport; mm = millimeter
