# Supplementary material for: Risk Factors for Contra-Lateral Secondary Anterior Cruciate Ligament Injury: A Systematic Review with Meta-Analysis
Source: Sports Med. 2021 Jan 30;51(7):1419–38. doi: 10.1007/s40279-020-01424-3 (PMC8222029; doi:10.1007/s40279-020-01424-3)
Supplement: Supplementary file 3 — (DOCX 60 KB) [file 40279_2020_1424_MOESM3_ESM.docx]

**Online resource 3**. Sub group meta-analyses of pediatric populations

**Males Females**

**I^2^ <0.001**

**Figure 1.** Sex differences in the odds of sustaining a C-ACL injury (C-ACL injury n=130, controls n=754). P=pediatric

**>14 years <14 years**

**I^2^ <0.001**

**Figure 2.** Difference in the odds of sustaining a C-ACL injury between those older than 14 and those younger than 14 years (C-ACL injury n=78, controls n=337)

**No family history Family history**

**I^2^ <0.001**

**Figure 3.** Difference in the odds of sustaining a C-ACL injury between those with a family history of anterior cruciate ligament injury and those without (C-ACL injury n=78, controls n=337). P=pediatric
