# Supplementary material for: Risk Factors for Contra-Lateral Secondary Anterior Cruciate Ligament Injury: A Systematic Review with Meta-Analysis
Source: Sports Med. 2021 Jan 30;51(7):1419–38. doi: 10.1007/s40279-020-01424-3 (PMC8222029; doi:10.1007/s40279-020-01424-3)
Supplement: Supplementary file 4 — (DOCX 39 KB) [file 40279_2020_1424_MOESM4_ESM.docx]

**Online resource 4.** Sensitivity analysis of meta-analytic findings

**Table 1.** Sensitivity analysis of meta-analytic findings

|  | **All studies** | | | **Atypical inclusion criteria (pediatric)** | | | **Atypical inclusion criteria (follow-up ≤ 2 years)** | | | **Findings with exclusion of studies** |
| --- | --- | --- | --- | --- | --- | --- | --- | --- | --- | --- |
| **Factor** | Studies  (n) | Participants  (n) | Odds ratio (95% CI) | Studies  (n) | Participants  (n) | Odds ratio (95% CI) | Studies  (n) | Participants  (n) | Odds ratio (95% CI) |  |
| Sex | 28 | 59 448 | 1.35  (1.14;1.61) | 23 | 58564 | 1.41  (1.18;1,69) | 21 | 38 361 | 1.21 (1.01;49) | No changes |
| Age (continuous variable) | 7 | 39 948 | 0.73  (0.59:0.90) | No studies removed | NA | NA | 5 | 20 880 | 0.56  (0.29;1.06) | Age no longer significant |
| Family history | 9 | 2 836 | 2.07  (1.54;2.80) | 7 | 2421 | 1.89  (1.38;2.59) | 8 | 2 603 | 2.13  (1.33;2.94) | No changes |
| Meniscal injury | 4 | 34 059 | 1.21  (1.03;1.42) | No studies removed | NA | NA | 3 | 31 676 | 1.22  (1.03;1.44) | No changes |
| Return to high activity level | 6 | 4 583 | 3.26  (2.10;5.06) | 5 | 4341 | 3.61  (2.09;6.22) | 5 | 2 200 | 3.83  (2.09;7.01) | No changes |

CI = confidence interval; NA = not applicable
