# Supplementary material for: Risk Factors for Contra-Lateral Secondary Anterior Cruciate Ligament Injury: A Systematic Review with Meta-Analysis
Source: Sports Med. 2021 Jan 30;51(7):1419–38. doi: 10.1007/s40279-020-01424-3 (PMC8222029; doi:10.1007/s40279-020-01424-3)
Supplement: Supplementary file 5 — (DOCX 112 KB) [file 40279_2020_1424_MOESM5_ESM.docx]

**Online resource 5. Quality index scores for all papers included in the systematic review and publication bias for sex as a risk factor for contralateral injury**

Table 1.

| **Quality scores** | *Reporting* | | | | | | | *External*  *validity* | | *Internal validity – Bias* | | | | *Internal validity –*  *confounding*  *(selection bias)* | | | | *Power* | **Total score** |
| --- | --- | --- | --- | --- | --- | --- | --- | --- | --- | --- | --- | --- | --- | --- | --- | --- | --- | --- | --- |
|  | Item | | | | | | | | | | | | | | | | | | |
| **Article** | **1** | **2** | **3** | **5** | **6** | **7** | **10** | **11** | **12** | **15** | **16** | **18** | **20** | | **21** | **22** | **25** | **27** |  |
| Allen et al. 2016 | 1 | 1 | 1 | 2 | 1 | 1 | 1 | 1 | 0 | 1 | 1 | 1 | 1 | | 1 | 1 | 1 | 1 | 17/19 = 89% |
| Annear et al. 2019 | 1 | 1 | 0 | 1 | 1 | 1 | 0 | 0 | 0 | 1 | 1 | 1 | 1 | | 1 | 1 | 0 | 0 | 11/19 = 58% |
| Bourke et al. 2012 | 1 | 1 | 0 | 1 | 1 | 1 | 1 | 1 | 1 | 1 | 1 | 1 | 1 | | 1 | 1 | 0 | 1 | 15/19 = 79% |
| Davey et al. 2019 | 1 | 1 | 1 | 1 | 1 | 1 | 1 | 1 | 1 | 1 | 1 | 1 | 2 | | 1 | 1 | 0 | 1 | 17/19 = 89% |
| Filbay et al. 2017 | 1 | 1 | 1 | 2 | 1 | 1 | 1 | 1 | 1 | 1 | 1 | 1 | 2 | | 1 | 0 | 1 | 0 | 17/19 = 89% |
| Fältström et al. 2013 | 1 | 1 | 1 | 0 | 1 | 1 | 1 | 0 | 0 | 1 | 1 | 1 | 1 | | 0 | 0 | 0 | 0 | 10/19 = 53% |
| fältström et al. 2016 | 1 | 1 | 1 | 1 | 1 | 1 | 1 | 1 | 1 | 1 | 1 | 1 | 1 | | 1 | 1 | 1 | 1 | 17/19 = 89% |
| Goshima et al. 2014 | 1 | 1 | 1 | 1 | 1 | 1 | 1 | 1 | 0 | 1 | 1 | 1 | 1 | | 1 | 1 | 0 | 0 | 14/19 = 74% |
| Grassi et al. 2020 | 1 | 1 | 1 | 1 | 1 | 1 | 1 | 1 | 1 | 1 | 1 | 1 | 1 | | 1 | 1 | 0 | 0 | 15/19 = 79% |
| Heath et al. 2019 | 1 | 1 | 1 | 1 | 1 | 1 | 1 | 1 | 1 | 1 | 1 | 1 | 1 | | 1 | 1 | 0 | 0 | 15/19 = 79% |
| Kaeding et al. 2015 | 1 | 1 | 1 | 0 | 1 | 1 | 1 | 1 | 1 | 1 | 1 | 1 | 1 | | 1 | 1 | 0 | 1 | 15/19 = 79% |
| Lai et al. 2018 | 1 | 1 | 0 | 1 | 1 | 1 | 1 | 1 | 0 | 1 | 1 | 1 | 1 | | 1 | 1 | 0 | 0 | 13/19 = 68% |
| Larsson et al. 2017 | 1 | 1 | 0 | 1 | 1 | 1 | 1 | 1 | 0 | 1 | 1 | 1 | 1 | | 1 | 1 | 0 | 1 | 14/19 = 74% |
| Levins et al. 2017 | 1 | 1 | 1 | 1 | 1 | 1 | 1 | 1 | 1 | 1 | 1 | 1 | 2 | | 1 | 1 | 1 | 0 | 17/19 = 89% |
| Leys et al. 2011 | 1 | 1 | 1 | 2 | 1 | 1 | 1 | 1 | 0 | 1 | 1 | 1 | 1 | | 1 | 1 | 0 | 0 | 15/19 = 79% |
| Magnusson et al. 2018 | 1 | 1 | 1 | 2 | 1 | 1 | 1 | 1 | 1 | 1 | 1 | 1 | 1 | | 1 | 1 | 1 | 1 | 18/19 = 95% |
| Maletis et al. 2015 | 1 | 1 | 0 | 1 | 1 | 1 | 1 | 1 | 0 | 1 | 1 | 1 | 1 | | 1 | 1 | 1 | 1 | 15/19 = 79% |
| Mardani-Kivi et al. 2019 | 1 | 1 | 1 | 0 | 1 | 1 | 1 | 1 | 0 | 1 | 1 | 1 | 1 | | 1 | 1 | 0 | 1 | 14/19 = 74% |
| McPherson et al. 2019 | 1 | 1 | 1 | 1 | 1 | 1 | 1 | 1 | 1 | 1 | 1 | 1 | 1 | | 1 | 1 | 0 | 0 | 15/19 = 79% |
| Mohtadi et al. 2016 | 1 | 1 | 1 | 1 | 1 | 1 | 1 | 1 | 1 | 1 | 1 | 1 | 1 | | 1 | 1 | 1 | 0 | 16/19 = 84% |
| Morgan et al. 2016 | 1 | 1 | 1 | 1 | 1 | 1 | 1 | 1 | 0 | 1 | 1 | 1 | 1 | | 1 | 1 | 0 | 0 | 14/19 = 74% |
| Nakase et al. 2012 | 1 | 1 | 1 | 0 | 1 | 1 | 1 | 0 | 0 | 1 | 1 | 1 | 1 | | 1 | 1 | 0 | 1 | 13/19 = 68% |
| Patel et al. 2019 | 1 | 1 | 1 | 2 | 1 | 1 | 1 | 1 | 1 | 1 | 1 | 1 | 1 | | 1 | 1 | 1 | 1 | 18/19 = 95% |
| Paterno et al. 2014 | 1 | 1 | 1 | 1 | 1 | 1 | 1 | 1 | 0 | 1 | 1 | 1 | 1 | | 1 | 1 | 1 | 0 | 15/19 = 79% |
| Paterno et al. 2018 | 1 | 1 | 1 | 1 | 1 | 1 | 1 | 0 | 0 | 1 | 1 | 1 | 1 | | 1 | 1 | 0 | 0 | 13/19 = 68% |
| Perkins et al. 2019 | 1 | 1 | 0 | 1 | 1 | 1 | 1 | 1 | 1 | 1 | 1 | 1 | 1 | | 1 | 1 | 1 | 0 | 15/19 = 79% |
| Pfeiffer et al. 2018 | 1 | 1 | 0 | 2 | 1 | 1 | 0 | 1 | 0 | 1 | 1 | 1 | 2 | | 1 | 1 | 1 | 1 | 16/19 = 84% |
| Pincewski et al. 2007 | 1 | 1 | 1 | 1 | 1 | 1 | 1 | 1 | 0 | 1 | 1 | 1 | 1 | | 1 | 1 | 0 | 0 | 14/19 = 74% |
| Pujol et al. 2007 | 1 | 1 | 1 | 0 | 1 | 1 | 1 | 1 | 1 | 1 | 1 | 1 | 1 | | 1 | 1 | 0 | 0 | 14/19 = 74% |
| Rosenstiel et al. 2019 | 1 | 1 | 1 | 1 | 1 | 1 | 1 | 1 | 1 | 1 | 1 | 1 | 1 | | 1 | 1 | 0 | 0 | 15/19 = 79% |
| Salmon et.al 2005 | 1 | 1 | 1 | 1 | 1 | 1 | 1 | 1 | 0 | 1 | 1 | 1 | 1 | | 1 | 1 | 1 | 0 | 15/19 = 79% |
| Salmon et.al 2006 | 1 | 1 | 1 | 0 | 1 | 1 | 1 | 1 | 1 | 1 | 1 | 1 | 2 | | 1 | 1 | 0 | 0 | 15/19 = 79% |
| Salmon et.al 2018 | 1 | 1 | 1 | 1 | 1 | 1 | 1 | 1 | 0 | 1 | 1 | 1 | 1 | | 1 | 1 | 0 | 0 | 14/19 = 74% |
| Schickendanz et.al 1993 | 1 | 1 | 0 | 0 | 1 | 1 | 1 | 0 | 0 | 1 | 1 | 1 | 1 | | 1 | 1 | 0 | 0 | 11/19 = 58% |
| Schmale et al. 2014 | 1 | 1 | 1 | 0 | 1 | 1 | 1 | 1 | 0 | 1 | 1 | 1 | 1 | | 1 | 1 | 0 | 0 | 13/19 = 68% |
| Shelbourne et al. 1998 | 1 | 1 | 1 | 1 | 1 | 1 | 1 | 1 | 1 | 1 | 1 | 1 | 1 | | 1 | 1 | 1 | 1 | 17/19 = 89% |
| Shelbourne et al. 2009 | 1 | 1 | 1 | 0 | 1 | 1 | 1 | 1 | 0 | 1 | 1 | 1 | 1 | | 1 | 1 | 0 | 1 | 14/19 = 74% |
| Souryal et al. 1988 | 1 | 1 | 0 | 0 | 1 | 0 | 1 | 1 | 0 | 1 | 1 | 1 | 0 | | 1 | 1 | 0 | 0 | 10/19 = 53% |
| Sousa et al. 2017 | 1 | 1 | 1 | 2 | 1 | 1 | 1 | 1 | 0 | 1 | 1 | 1 | 1 | | 1 | 1 | 1 | 1 | 17/19 = 89% |
| Thompson et al. 2015 | 1 | 1 | 1 | 1 | 1 | 1 | 1 | 1 | 0 | 1 | 1 | 1 | 1 | | 1 | 1 | 1 | 0 | 15/19 = 79% |
| Wasserstein et al. 2013 | 1 | 1 | 0 | 1 | 1 | 1 | 1 | 1 | 1 | 1 | 1 | 1 | 1 | | 1 | 1 | 1 | 1 | 16/19 = 84% |
| Webb et al. 2013 | 1 | 1 | 0 | 1 | 1 | 1 | 1 | 1 | 1 | 1 | 1 | 1 | 2 | | 1 | 1 | 1 | 0 | 16/19 = 84% |
| Webster et al. 2014 | 1 | 1 | 1 | 1 | 1 | 1 | 1 | 1 | 1 | 1 | 1 | 1 | 1 | | 1 | 1 | 0 | 0 | 16/19 = 84% |
| Wright et al. 2007 | 1 | 1 | 0 | 0 | 1 | 0 | 1 | 1 | 1 | 1 | 1 | 1 | 1 | | 1 | 1 | 0 | 0 | 12/19 = 63% |

**Fig 1.** Funnel plot with trim and fill imputations for sex difference for the risk of sustaining a contralateral anterior cruciate ligament injury
